# Targeting Cannabinoid Signaling in the Immune System: “High”-ly Exciting Questions, Possibilities, and Challenges

**DOI:** 10.3389/fimmu.2017.01487

**Published:** 2017-11-10

**Authors:** Attila Oláh, Zoltán Szekanecz, Tamás Bíró

**Affiliations:** ^1^Department of Physiology, Faculty of Medicine, University of Debrecen, Debrecen, Hungary; ^2^Department of Internal Medicine, Division of Rheumatology, Faculty of Medicine, University of Debrecen, Debrecen, Hungary; ^3^Department of Immunology, Faculty of Medicine, University of Debrecen, Debrecen, Hungary

**Keywords:** cannabinoid signaling, endocannabinoid, inflammation, immune response, phytocannabinoid, multiple sclerosis, tumor immunology, marijuana

## Abstract

It is well known that certain active ingredients of the plants of *Cannabis* genus, i.e., the “phytocannabinoids” [pCBs; e.g., (−)-*trans*-Δ^9^-tetrahydrocannabinol (THC), (−)-cannabidiol, etc.] can influence a wide array of biological processes, and the human body is able to produce endogenous analogs of these substances [“endocannabinoids” (eCB), e.g., arachidonoylethanolamine (anandamide, AEA), 2-arachidonoylglycerol (2-AG), etc.]. These ligands, together with multiple receptors (e.g., CB_1_ and CB_2_ cannabinoid receptors, etc.), and a complex enzyme and transporter apparatus involved in the synthesis and degradation of the ligands constitute the endocannabinoid system (ECS), a recently emerging regulator of several physiological processes. The ECS is widely expressed in the human body, including several members of the innate and adaptive immune system, where eCBs, as well as several pCBs were shown to deeply influence immune functions thereby regulating inflammation, autoimmunity, antitumor, as well as antipathogen immune responses, etc. Based on this knowledge, many *in vitro* and *in vivo* studies aimed at exploiting the putative therapeutic potential of cannabinoid signaling in inflammation-accompanied diseases (e.g., multiple sclerosis) or in organ transplantation, and to dissect the complex immunological effects of medical and “recreational” marijuana consumption. Thus, the objective of the current article is (i) to summarize the most recent findings of the field; (ii) to highlight the putative therapeutic potential of targeting cannabinoid signaling; (iii) to identify open questions and key challenges; and (iv) to suggest promising future directions for cannabinoid-based drug development.

## Introduction

### The Endocannabinoid System (ECS) and Its Connections in a Nutshell

**Figure 1:**
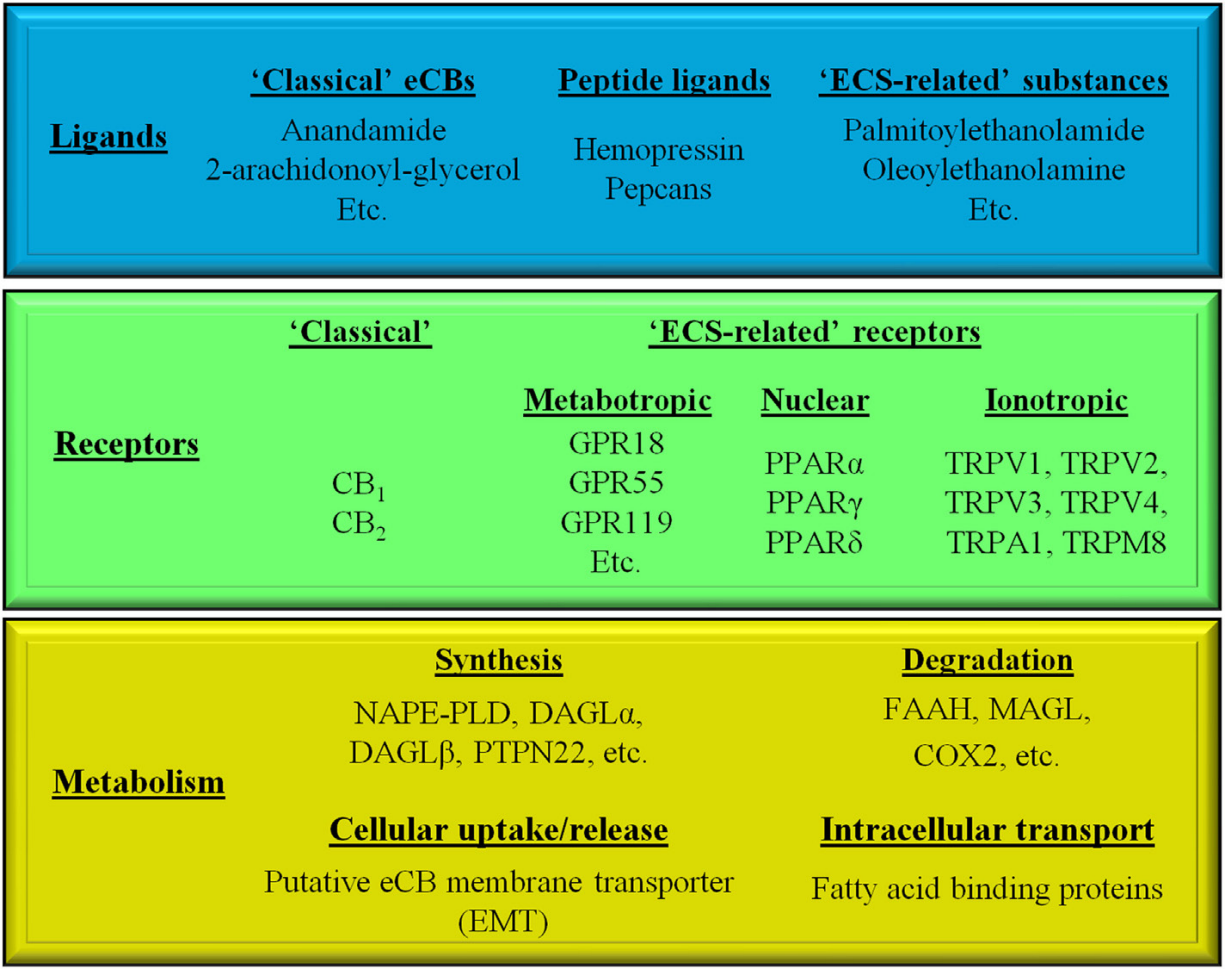
Simplified overview of the endocannabinoid system.

The ECS is a recently emerging, multifaceted signaling system, comprising various endogenous ligands [i.e., the “endocannabinoids” (eCBs), e.g., arachidonoylethanolamine (a.k.a. anandamide, AEA), 2-arachidonoylglycerol (2-AG), etc.], eCB-responsive receptors (e.g., CB_1_ and CB_2_ cannabinoid receptors, etc.), as well as enzymes and transporters involved in the synthesis [e.g., N-acyl phosphatidylethanolamine-specific phospholipase D (NAPE-PLD), diacylglycerol lipase-α and -β, protein tyrosine phosphatase non-receptor type 22 (PTPN22), etc.], cellular uptake/release [i.e., the putative endocannabinoid membrane transporter (EMT)], intracellular transport (various fatty acid-binding proteins) and degradation [e.g., fatty acid amide hydrolase (FAAH), monoacylglycrol lipase, cyclooxygenase 2 (COX2), etc.] of the eCBs (Figure 1) (1–10).

Moreover, in a wider sense, based on structural similarities and/or partial overlaps in the ligand affinities, many other receptors [e.g., the recently deorphanized metabotropic GPR18, GPR55, and GPR119, the intranuclear peroxisome proliferator-activated receptors (PPARs), as well as several members of the transient receptor potential (TRP) ion channel superfamily] together with some of their endogenous ligands (e.g., palmitoylethanolamide, oleoylethanolamine) can be classified as “ECS-related” entities (Figure 1) (11–14).

Besides the high number of ligands and potential receptors, complexity of the cannabinoid signaling is further increased by two phenomena: (i) the often observed biased agonism (i.e., when the same receptor exhibits signaling preference among its possible second messenger pathways depending on the actual ligand) of the metabotropic cannabinoid receptors (15, 16) and (ii) by their potential of forming heteromers either with each other, or with various other G protein-coupled receptors.

**Figure 2:**
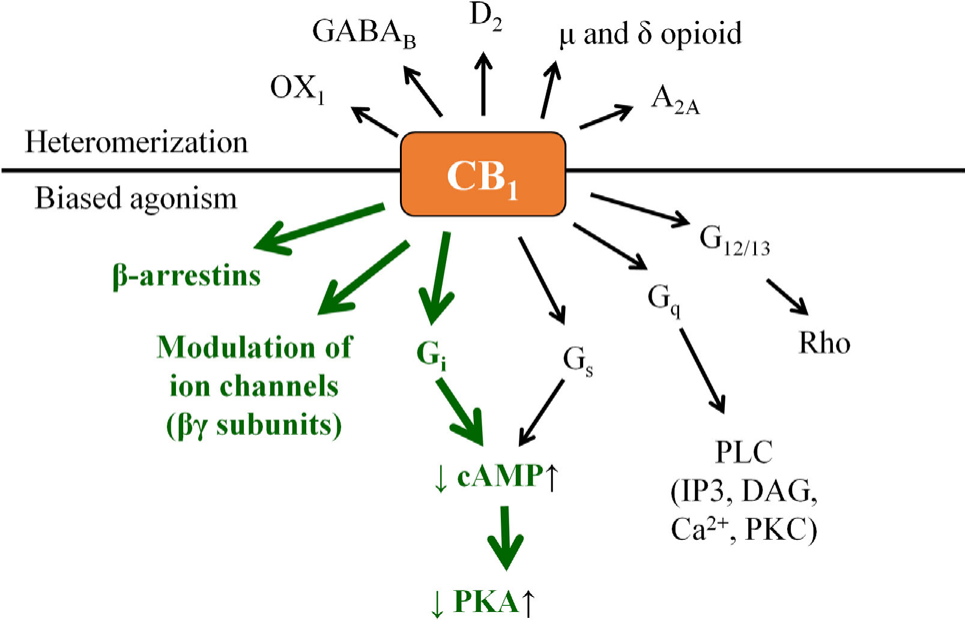
“Classical” CB_1_ signaling (green) and beyond: examples for biased agonism and heteromerization. Note that besides the presented complexity, actual biological action of a given CB_1_ modulator may also depend on its capability to penetrate through the cell membrane. Theoretically, cell-penetrating agonists/antagonists (i.e., ones being capable to act on both the surface membrane and mitochondrial CB_1_) and their extracellularly restricted variants (i.e., ones targeting exclusively the surface membrane subset of CB_1_) may also exert differential biological actions; however, such comparisons remain to be performed in future targeted studies.

Indeed, CB_1_ is generally considered to signal through G_αi_ protein leading to a decrease in the intracellular cyclic adenosine monophosphate (cAMP) level, and activation of the β-arrestin 1 and 2 pathways. On one hand, signaling bias can lie in the “choice” of the ligand in preferring (i.e., activating with higher relative efficiency) the β-arrestin pathway(s) over the G protein-coupled one or vice versa. On the other hand, in some cases coupling to G_αs_, G_αq_, or G_α12/13_ proteins (leading to the elevation of cAMP level, activation of phospholipase C, or Rho pathway, respectively) was also observed, adding an extra layer of complexity to CB_1_ signaling (Figure 2) (14–21). Importantly, similar to many other G protein-coupled receptors, biased signaling was described in case of CB_2_ or even in case of the “ECS-related” GPR18, GPR55, and GPR119 (14, 16, 19–22).

Moreover, without being exhaustive, CB_1_ was shown to form functional heteromers with, e.g., δ opioid receptor (DOR) (23), A_2A_ adenosine receptor (24), D_2_ dopamine receptor (25), orexin-1 receptor (26), etc., whereas CB_2_ was proven to heteromerize with, e.g., CXCR4 chemokine receptor (27), or GPR55 (28). On top of that, functional cooperation between CB_1_ and several receptor tyrosine kinases was also observed [Figure 2; extensively reviewed in Ref. (19)].

Considering the above described complexity of the system, it is not surprising that, at least some parts of, the ECS is present in virtually every cell type of the human body, and it was shown to be involved in the regulation of a plethora of physiological processes. In the central nervous system (CNS), these processes include regulation of appetite, pain sensation, mood, and memory, whereas in the peripheral tissues, e.g., bone formation, spermatogenesis, sebum production, etc., and, maybe most importantly, immune functions (7, 29–37). Indeed, eCB signaling was shown to be an important orchestrator of both the innate and adaptive immune responses. Although there are some contradictions in the literature, ECS is generally considered to be a homeostatic “gate-keeper” of the immune system, preventing the onset of pathological, overwhelming proinflammatory responses. For example, CB_1_ and CB_2_ double KO mice exhibited stronger allergic inflammation than wild-type mice. Importantly, these alterations were shown to be mediated *via* the pathologically increased chemokine release of epidermal keratinocytes, suggesting that lack of homeostatic eCB signaling skewed keratinocytes toward a proinflammatory phenotype. On the other hand, FAAH^−/−^ mice (having elevated eCB levels) showed reduced allergic responses (29), further arguing for the concept that elevation of the eCB tone (e.g., by abrogating degradation of the eCBs or by directly activating cannabinoid receptors) usually leads to potent anti inflammatory/antiallergic actions [extensively reviewed in, e.g., Ref. (7, 30, 31, 33, 34, 36–41)].

### Active Components of *Cannabis sativa* (Hemp)—Phytocannabinoids (pCBs) and Beyond

**Figure 3:**
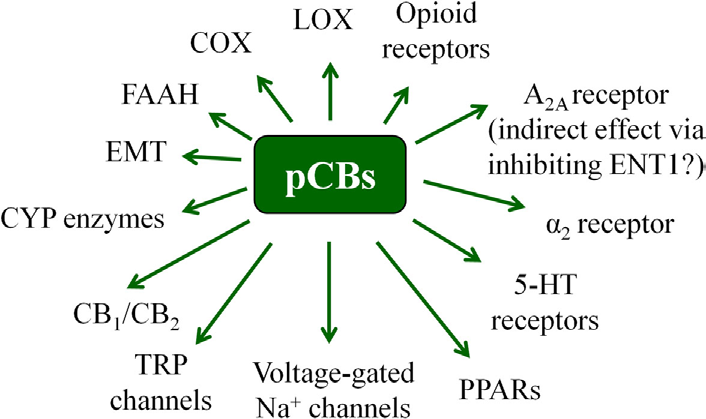
Overview of the most important potential targets of the pCBs. Note that there are more than 100 pCBs in *Cannabis sativa*, and each of them can be characterized by a unique “molecular fingerprint.” Obviously, every pCB is evidenced to interact with only a part of the potential targets presented on the figure. Moreover, the interactions can often lead to opposing molecular biology outcomes [e.g., THC is a partial CB_1_ agonist, whereas CBD is a CB_1_ antagonist/inverse agonist].

It is known since ancient times that consumption of different parts of the plant *Cannabis sativa* can lead to psychotropic effects. Moreover, mostly, but not exclusively because of its potent analgesic actions, it was considered to be beneficial in the management of several diseases (19, 42, 43). Nowadays it is a common knowledge that these effects were mediated by the complex mixture of biologically active substances produced by the plant. So far, at least 545 active compounds have been identified in it, among which, the best-studied ones are the so-called pCBs. It is also noteworthy that besides these compounds, ca. 140 different terpenes [including the potent and selective CB_2_ agonist sesquiterpene β-caryophyllene (BCP) (44)], multiple flavonoids, alkanes, sugars, non-cannabinoid phenols, phenylpropanoids, steroids, fatty acids, and various nitrogenous compounds (19, 45, 46) can be found in the plant, individual biological actions of which are mostly still nebulous. Among the so far identified > 100 pCBs (19, 47), the psychotropic (−)-*trans*-Δ^9^-tetrahydrocannabinol (THC) and the non-psychotropic (−)-cannabidiol (CBD) are the best-studied ones, exerting a wide-variety of biological actions [including but not exclusively: anticonvulsive, analgesic, antiemetic, and anti inflammatory effects, etc.; for extensive reviews, see e.g., Ref. (8, 19)]. Of great importance, pCBs have been shown to modulate the activity of a plethora of cellular targets, extending their impact far beyond the “classical” (see above) cannabinoid signaling. Indeed, besides being agonists [or in some cases even antagonists! (48)] of CB_1_ and CB_2_ cannabinoid receptors, some pCBs were shown to differentially modulate the activity of certain TRP channels, PPARs, serotonin, α adrenergic, adenosine or opioid receptors, and to inhibit COX and lipoxygenase enzymes, FAAH, EMT, etc. (8, 19, 48, 49). Moreover, from a clinical point-of-view, it should also be noted that pCBs can indirectly modify pharmacokinetics of multiple drugs (e.g., cyclosporine A) by interacting with several cytochrome P 450 (CYP) enzymes (50, 51). Taken together, pCBs can be considered as multitarget polypharmacons, each of them having unique “molecular fingerprints” created by the characteristic activation/inhibition pattern of its locally available cellular targets (Figure 3) (52).

## Selected Episodes of “(Phyto)Cannabinoid Immunobiology”

As we briefly mentioned above, ECS is generally considered to be one of the “gate-keepers” of the immune system, preventing the onset of pathological immune responses [extensively reviewed in Ref. (7, 30, 31, 33, 34, 36–41)]. Considering that many of the aforementioned “non-classical” cannabinoid targets (e.g., TRP channels and PPARs) are also key regulators of the immune system (53–55), it is not surprising that both eCBs and pCBs can deeply influence immune responses. Based on this, elevation of the eCB tone and/or application of pCBs augur to be beneficial in those conditions, when one needs to suppress potentially detrimental immune responses (e.g., in organ transplantation or autoimmune diseases, etc.). However, administration of such medications may hold the risk of suppressing desired immunological reactions against pathogens and tumor cells.

Since clinical administration of medical marijuana as well as of purified/synthetic pCBs is nowadays under increasing scrutiny, in the next chapters we intend to summarize the most important data of the field, and, while also highlighting existing controversies and limitations, to point toward possible future directions of cannabinoid-based medicines (Table 1).

**Table 1:** Overview of the compounds mentioned in the manuscript.

Compound	Model system	Mechanism		Phenomenon	Reference
		Receptors/pathway	Involved cell type/tissue		
AEA	Human	-	Lymphocytes	Elevation in MS patients	(82, 83)
AEA	TMEV	CB_1_	TMEV-infected astrocytes	Increased IL-6 release	(86)
2-AG	Acute and chronic EAE	?	M2 macrophages, lymphocytes	Direct and immune-mediated neuroprotection	(88)
THC	WT and CB_1_/_2_ KO mice	CB_1_	T cells	Delayed rejection of skin graft, reduced T cell proliferation, IL-2 and IFN-γ secretion	(62)
THC	Murine acute GVHD model	CB_1_ < CB_2_	Effector T cells, Foxp3^+^ Treg cells	Reduced weight loss, intestinal tissue injury and mortality	(64)
THC	Relapsing EAE in ABH mice	?	?	Slower accummulation of disability	(117)
THC	4T1 mammary carcinoma cell line	CB_2_	Complex actions	Increased metastasis formation *in vivo* due to the inhibition of the antitumor immune response	(140)
THC	C57Bl/6 mice	CB_1_/CB_2_-dependent and independent mechanisms	Splenocytes	Enhanced HIV antigen-specific immune response	(156)
THC	*Legionella pneumophila* infection	CB_1_/CB_2_	T cells	Th1 → Th2 shift (CB_1_: suppression of IL-12Rβ2; CB_2_: enhancement of GATA-3 upregulation)	(163)
CBD	Mouse autoimmune myocarditis	Decreased oxidative/nitrative stress	T cells	Attenuated CD3^+^ and CD4^+^ response, myocardial fibrosis and dysfunction	(63)
CBD	Human phase II clinical trial (NCT01385124)	?	?	Oral CBD improved standard GVHD prophylaxis	(65)
CBD	Relapsing EAE in ABH mice	Voltage-gated Na^+^ channels	?	Slower accumulation of disability	(117)
CBD	EAE	?	T cells	T cell exhaustion, decreased antigen presentation, antiproliferative, and antioxidant effects	(119)
CBD	TMEV-induced demyelinating disease	A_2A_ (?) (maybe via inhibiting ENT1?)	Endothelial cells, leukocytes	Decreased leukocyte transmigration	(120, 123)
BCP	EAE	CB_2_	Microglia, CD4^+^ and CD8^+^ T cells, Th1/Treg balance	Suppression of motor paralysis and neuroinflammation	(102)
VCE-003 (CBG-derivative)	EAE	CB_2_ and PPARγ	T cells, macrophages	Amelioration of neurological defects; inhibition of Th1/Th17 cytokine/chemokine secretion, and M1 polarization	(101)
CB52	EAE	CB_1_	Microglia, T cell, oligodendrocyte	Reduced microglia activation, nitrotyrosine formation, T cell infiltration, oligodendrocyte toxicity, myelin loss, and axonal damage in the mouse spinal cord white matter	(87)
Gp1a	EAE	CB_2_	Th1/Th17	Amelioration of EAE, reduction of Th17 differentiation	(91)
Gp1a	CLP	CB_2_	Neutrophil granulocytes	Decreased neutrophil recruitment, but increased activation; decreased serum IL-6 level, bacteriemia and lung damage	(145)
R(+)WIN55,212	Mouse Chagas disease model	CB_1_ (?)	Cardiomyocytes	Reduced invasion of cardiomyoblasts, increased parasitemia	(146)
SR144528	EAE	CB_2_ antagonism	Spinal cord, splenic mononuclear cells	Worsening of clinical severity	(90)
SR144528	Experimental cerebral malaria (ECM)	CB_2_ antagonism	CD11b^+^ macrophages and neutrophils (?)	Increased ECM resistance	(147)
AM630 and JTE907	Mice	CB_2_ inverse agonism	Acitvated lymph nodes	Improved antigen-specific immune response	(149)

### Organ Transplantation

In order to improve quality of life and life expectancy, prevention of acute and chronic rejection following solid organ transplantation, and avoidance of the development of *graft-versus-host* disease (GVHD) after bone marrow transplantation are key clinical challenges. Indeed, in order to overcome these problems, a number of different promising therapeutic opportunities are currently investigated, including, e.g., transplantation of tolerogenic dendritic cells (DCs) (56), modulation of myeloid-derived suppressor cells (MDSCs) (57) or regulatory T cells (Treg) (58), as well as inhibition of Janus kinase signaling (59), etc. Due to their well-described anti inflammatory effects, pCBs are also potential candidates to improve therapeutic protocols of transplantation (60).

The concept that positive modulation of cannabinoid signaling could be helpful in transplantation is supported by multiple pieces of evidence. Indeed, recent findings showed that cardiac allograft rejection was accelerated in CB_2_^−/−^ mice compared to wild-type recipients. In these experiments, bone marrow-derived dendritic cells (BM-DCs) of CB_2_^−/−^ mice exhibited enhanced secretion of the proinflammatory cytokines interleukin (IL)-1β, IL-6, and tumor necrosis factor, as well as transforming growth factor-β1 upon Toll-like receptor (TLR) activation by lipopolysaccharide (LPS) or CpG. In addition, secretion of the Th1/Th17-promoting IL-12 and IL-23 cytokines was also increased in CB_2_^−/−^ BM-DCs, and CD4^+^ T cells of the KO mice showed an enhanced capacity to differentiate into interferon (IFN)-γ- or IL-17-producing effector cells, altogether suggesting that CB_2_ may be a potential therapeutic target in the clinical management of *host-versus-graft* reactions (61).

Importantly, not only CB_2_, but also CB_1_ emerged to be a promising therapeutic target in preventing organ rejection. Indeed, in mice, THC was recently found to attenuate *host-versus-graft* response and delayed rejection of skin graft by (i) reducing T cell proliferation and activation in draining lymph nodes of the recipient mice and (ii) decreasing early stage rejection-indicator cytokines, including IL-2 and IFN-γ. Moreover, by employing selective antagonists, as well as CB_1_ and CB_2_ KO mice, the authors also showed that these effects were mediated *via* inducing MDSCs and activation of CB_1_ receptors (62).

Moreover, another study showed that, besides THC, CBD may also represent a promising novel treatment modality in organ transplantation (63), and administration of certain pCBs appeared to be promising in GVHD, too. Indeed, in an acute murine model of GVHD, THC (20 mg/kg i.p.) suppressed splenocyte transfer-induced weight loss, liver and intestinal tissue injury, as well as mortality. Importantly, THC treatment (i) reduced the expansion of donor-derived effector T cells; (ii) blocked the killing of host-derived immune cells; (iii) promoted Foxp3^+^ Treg cells; and (iv) normalized the impaired hematopoiesis seen during GVHD. The effects were thought to be CB_1_- and CB_2_-mediated ones, since both CB_1_ (AM251) and CB_2_ (SR144528) specific inverse agonists were able to partially prevent the effect of THC in normalizing splenomegaly. Among them the CB_2_-selective SR144528 appeared to be more efficient, and the combination of the two inverse agonists could completely abrogate THC’s beneficial effects arguing for that targeting CB_1_ and/or CB_2_ cannabinoid receptors may constitute a novel treatment modality against acute GVHD (64). Finally, data of a recent phase II clinical study (reference ID at clinicaltrials.gov: NCT01385124) showed that orally administered CBD (300 mg/day) is a safe and efficient way to improve the standard GVHD prophylaxis (65).

### Autoimmune Diseases

The concept that dysregulation of the ECS can play a role in autoimmune diseases is supported by several lines of evidence. Indeed, the missense Arg → Trp (R620W) polymorphism of the eCB synthesizing enzyme PTPN22 [encoding lymphoid protein tyrosine phosphatase (LYP), which is important in negatively controlling activation of T lymphocytes] was found to be associated with increased risk of type 1 diabetes mellitus (T1DM), rheumatoid arthritis (RA), juvenile idiopathic arthritis, systemic lupus erythematosus, Graves disease, myasthenia gravis, generalized vitiligo, and granulomatosis with polyangiitis [previously known as Wegener’s granulomatosis; reviewed in Ref. (66)]. Moreover, a common dinucleotide polymorphism of CB_2_, resulting in a Gln → Arg substitution (Q63R), which is accompanied by a reduced capability of CB_2_-mediated signaling to suppress T cell proliferation (67), was associated with an increased risk of immune thrombocytopenia (68), and celiac disease (69). In line with these data, cooccurrence of Q63R polymorphism and immune-mediated disorders in chronic hepatitis C virus (HCV) infection was also observed (70), whereas the healthy CB_2_ variant was associated with more severe inflammation and hepatocellular necrosis, most probably because the intact CB_2_ could more efficiently inhibit antiviral T cell functions (71). Thus, the concepts to positively modulate eCB tone, to activate CB_2_ receptor and to administer certain pCBs has already been suggested in, e.g., RA, T1DM, autoimmune myocarditis, ulcerative colitis, Crohn’s disease, etc. as well (63, 72–81), but so far, the “cannabinoid-wise” best explored autoimmune disease is unambiguously the multiple sclerosis (MS).

Indeed, AEA levels of the peripheral lymphocytes of MS patients was found to be elevated compared to healthy individuals, suggesting the development of a complex dysregulation in the ECS of MS patients (82, 83). Moreover, TLR and cannabinoid receptor cross-talk (84), as well as novel, “ECS-related” receptors (GPR18 and GPR55) have all been suggested to play a role in the pathogenesis of MS (85), and CB_1_ (86–88), but especially CB_2_ also emerged as a remarkably powerful and multifaceted future therapeutic target in this disease (88–100).

In line with these data, daily administration of compound VCE-003 [a quinone derivative of the non-psychotropic pCB (−)-cannabigerol (CBG)] from day 6 postimmunization for 21 days was able to ameliorate the neurological defects and the severity of experimental autoimmune encephalomyelitis (EAE; a murine model of MS) induced by subcutaneous immunization with myelin oligodendrocyte glycoprotein (MOG_35–55_; 300 µg) and *Mycobacterium tuberculosis* (200 µg) in a 1:1 mix with incomplete Freund’s adjuvant in mice. VCE-003 inhibited the secretion of Th1/Th17 cytokines and chemokines in primary murine T cells and dampened the IL-17-induced, proinflammatory M1 polarization of macrophages in a CB_2_ and PPARγ-dependent manner (101). In line with these data, BCP suppressed motor paralysis and neuroinflammation in EAE by inhibiting microglial cells, CD4^+^ and CD8^+^ T lymphocytes, as well as protein expression of proinflammatory cytokines. Furthermore, it diminished axonal demyelination and modulated Th1/Treg immune balance through the activation of CB_2_ (102).

With respect to the “classical” pCBs, beneficial effects of Sativex^®^ (a buccal spray, which contains THC and CBD in a 1:1 ratio) and other pCB-based formulations in alleviating symptoms (e.g., spasticity, sleeping difficulty, neurogenic lower urinary tract dysfunction, gait, etc.) of MS are also well-described (103–111). However, since there are some controversies in the available data (112, 113), their long-term efficiency needs to be further investigated (108, 114). The necessity of such studies is further underscored by the findings of a recently conducted, small clinical trial, in which Bedrocan^®^ (medical-grade cannabis, practically lacking CBD) was found to be effective in alleviating spasticity in 85% of Nabiximols (United States Adopted Name of Sativex^®^) non-responder patients (115), highlighting how deeply the exact composition of each pCB-based medication can influence the clinical efficacy.

Importantly, a growing body of evidence supports the concept that, besides providing symptomatic relief, treatment with appropriately selected pCBs may even have therapeutic value in MS. Indeed, an early study demonstrated that in EAE, THC-treated animals had either no or mild clinical symptoms with a survival greater than 95%, whereas more than 98% of the animals died in the placebo group. The better survival was accompanied by a marked reduction of inflammation in the CNS of THC-treated animals (116). Partially in line with these data, in a 3-year, phase III clinical trial (albeit the authors did not detect a beneficial effect of oral THC in progressive MS in general) a thorough subgroup analysis of people with less disability and more rapid progression demonstrated a significant deceleration of disease development in the oral THC group compared to placebo (117).

In another study, synthetic CBD could slow down the accumulation of disability from the inflammatory penumbra during relapsing EAE in ABH mice, possibly *via* blocking voltage-gated Na^+^ channels. In addition, non-sedating doses of THC dose-dependently inhibited the accumulation of disability during EAE (117). According to another EAE study, in which CBD was applied after the development of the disease, CBD (10 mg/kg mouse, i.p.) reversed EAE-induced downregulation of the phosphoinositide 3-kinase, protein kinase B (Akt), and mammalian/mechanistic target of rapamycin (mTOR) in the spinal cord. Moreover, CBD increased brain-derived neurotrophic factor level, downregulated IFN-γ and IL-17, upregulated PPARγ, and was found to promote neuronal survival by inhibiting c-Jun N-terminal kinase and p38 mitogen-activated protein kinase (118). Furthermore, another group demonstrated that in EAE, CBD exerted its immunoregulatory effects in activated MOG_35–55_-specific memory T cell cells *via* (i) suppressing proinflammatory Th17-related transcription; (ii) promoting T cell exhaustion/tolerance; (iii) enhancing IFN-dependent antiproliferative program; (iv) hampering antigen presentation; and (v) inducing antioxidant milieu resolving inflammation (119).

In line with the above data, CBD was found to be protective in Theiler’s encephalomyelitis virus (TMEV)-induced demyelinating disease (a viral model of MS) *via* activating A_2A_ receptors (120). It is noteworthy that although A_2A_ receptor most probably does not bind CBD, the phenomenon that it can mediate anti inflammatory actions of this pCB is not unprecedented. Indeed, similar effects were shown in murine acute lung injury model (121) and in human sebocytes (122) too, and they were thought to be realized *via* the inhibition of equilibrative nucleoside transporter(s) (e.g., ENT1, which mediates adenosine uptake of the cells) and the subsequently elevated “adenosine tone” (123).

### Tumor Immunology

Besides that medical marijuana is increasingly used in various tumors as a palliative treatment option (124), exploitation of the putative antitumor therapeutic potential of the endo- and pCBs is a hot topic of today’s cannabinoid research. *Via* activating CB_1_, CB_2_, or other cellular targets, both endo- and pCBs were already convincingly shown to exert complex [e.g., antiproliferative and proapoptotic effects, inhibition of angiogenesis, inhibition of tumor cell chemotaxis *via* activating CB_2_/CXCR4 heteromers, etc. (27, 125–134)] antitumor effects in most of the test systems *in vitro*. Although there are some exceptions [e.g., engagement of CB_1_ and CB_2_ were found to promote tumor progression in human melanoma cells (CB_1_), in renal cell carcinoma (CB_1_), as well as in colon cancer (CB_2_) (135–137)], most studies agree that their putative beneficial antitumor effects deserves further scrutiny (138, 139). However, several lines of evidence argue for that promising *in vitro* antitumor data cannot necessarily be translated to *in vivo* clinical benefits, because of the cannabinoid-mediated suppression of the antitumor (Th1-dominated) immune response (138, 139). Indeed, by investigating human (MCF-7 and MDA-MB-231) and mouse (4T1) mammary carcinoma cell lines expressing low to undetectable levels of CB_1_ and CB_2_, McKallip et al. found that these cells were not only resistant to THC-induced cytotoxicity, but THC treatment led to elevated 4T1 tumor growth and metastasis due to CB_2_-mediated inhibition of the antitumor immune response (140). Thus, although the very few available human studies [reviewed in Ref. (139)] suggest that THC and cannabis-extracts may have some beneficial effects beyond mere palliation, well-controlled, further studies are invited to find the most appropriate place of cannabinoid medications in the antitumoral therapeutic repertoire.

### Defense against Pathogens

Theoretically, administration of endo- and pCBs may hold the risk of dampening appropriate immune responses, and thereby increasing susceptibility toward infectious diseases. However, in light of the literature data, the situation appears to be more complex. Indeed, certain control over the overwhelming inflammatory processes in, e.g., systemic inflammatory response syndrome or sepsis would be undoubtedly highly desirable (141–143). Within the “classical” ECS, the anti inflammatory CB_2_ appears to be the most promising candidate to adjust such immune responses, but recently other receptors (e.g., CB_1_ or the apparently rather proinflammatory GPR55) were also proven to be potent and relevant regulators. The available (somewhat controversial) data about the roles of these receptors in sepsis, and especially the possible therapeutic exploitation of GPR55-antagonism in such conditions, is extensively reviewed in Ref. (34).

With respect to CB_2_, it has recently been shown that loss of homeostatic CB_2_ signaling worsened LPS-induced sepsis in mice, whereas activation of CB_2_ was proven to be beneficial *via* reducing leukocyte endothelial interactions, and thereby preventing further inflammatory damage (144). Similarly, in a cecal ligation and puncture (CLP) model of sepsis, CB_2_^−/−^ mice exhibited higher serum IL-6 levels and bacteremia, and had decreased survival rates, whereas CB_2_ agonism increased the mean survival time in wild-type mice (145). Furthermore, in a mice model of *Trypanosoma cruzi* infection (Chagas disease) it was shown that the non-specific CB receptor agonist R(+)WIN55,212 significantly reduced cardiac inflammation. However, it also led to considerably increased parasitemia, therefore therapeutic value of such non-specific drugs remained questionable (146).

In contrast to the aforementioned findings, in a mice model of cerebral malaria (a severe and often fatal complication of *Plasmodium falciparum* infection), CB_2_ *antagonism*, as well as the CB_2_^−/−^ genotype were protective, and led to enhanced survival and a diminished blood-brain barrier disruption (147). Moreover, CB_2_^−/−^ (but not CB_1_^−/−^) mice were resistant to LPS-driven suppression of serum progesterone levels and preterm birth (148). Last, but not least, transient administration of the CB_2_ inverse agonists AM630 (10 mg/kg) or JTE907 (3 mg/kg) during immunization was found to improve antigen-specific immune responses in young and aged mice through the upregulation of immunomodulatory genes in secondary lymphoid tissues (149).

Thus, in light of the above data, it seems to be highly likely that both enhancement and suppression of the eCB signaling might have therapeutic value in carefully selected clinical conditions, which already suggests that administration of pCBs and/or other cannabis-derivatives is also not without controversies. Indeed, although several pCBs were shown to exert potent direct antibacterial activity (150), their aforementioned immunosuppressive effects definitely limit their therapeutic administration in infections. Since excellent overviews of the effects of pCBs and the ECS in viral (151), and other infections (34) were published recently, here we will only highlight some of the most interesting controversies of the field.

Without being exhaustive, in a Wistar rat model of pneumococcal meningitis, CBD (10 mg/kg, i.p.) reduced host immune response, and prevented cognitive impairments (152). Chronic administration of THC induced intestinal anti-inflammatory miRNA expression during acute *Simian Immunodeficiency Virus* (SIV) infection of rhesus macaques (153), and did not increase viral load in brain tissue (154). Likewise, another study also showed that chronic THC administration did not increase viral load or aggravate morbidity; in contrast, it could actually ameliorate SIV disease progression, *via* retention of body mass, and attenuation of inflammation (155). Moreover, it was also shown that under certain conditions, THC could even enhance *Human Immunodeficiency Virus* (HIV) antigen-specific immune responses, which occurred through both CB_1_/CB_2_-dependent and -independent mechanisms (156), and findings showed no evidence for a negative effect of cannabis use on circulating CD4^+^ T cell counts/percentages in HCV-HIV coinfected patients (157). Thus, it is not surprising that medical marijuana is part of HIV/AIDS adjuvant treatment in several countries (158).

With respect to other infections, it is noteworthy that CBD was recently suggested to be explored as a treatment for individuals suffering from post-Ebola syndrome (159). Moreover, although it had no effect on *Hepatitis B Virus*, 10 µM CBD inhibited HCV replication by 86.4% *in vitro* (160). Finally, CBD (30 mg/kg/day, i.p.) increased survival, and promoted rescue of cognitive function in a murine model of cerebral malaria (161).

Despite these promising findings, data of some other studies argue against the administration of pCBs in infectious diseases. Indeed, chronic THC treatment decreased the efficacy of the memory immune response to *Candida* infection (162). In *Legionella pneumophila* infection, THC treatment prior to contamination induced a shift from Th1 to Th2 immunity in a CB_1_ and CB_2_ dependent manner (163). Moreover, THC impaired the inflammatory response to influenza infection (164), suppressed immune function, and enhanced HIV replication in a mice model, where human peripheral blood leukocytes (PBLs) were implanted into severe combined immunodeficient mice (huPBL-SCID mice) (165). Interestingly, investigation of plasmocytoid dendritic cells (pDCs) revealed an intriguing functional heterogeneity of the pCBs, i.e., THC (but not CBD!) suppressed secretion of IFN-α by pDC from both healthy and HIV^+^ donors, arguing for that although THC may impair antiviral responses, but this may also be protective in neuroinflammation associated with prolonged HIV infection (166). Taken together, these data suggest that although cannabinoid signaling may decrease the efficiency of certain antipathogen immune responses, in some cases it might still be beneficial by limiting overwhelming inflammatory response and tissue destruction. Further studies are therefore invited to determine and exploit exact therapeutic value of eCBs and pCBs in such diseases.

## Complex Immunological Effects of Medical and “Recreational” Marijuana Consumption

Considering the wide-spread popularity of marijuana consumption and the social debate about its legislative status, it is clear that there is an emerging demand from the scientific community, the society, as well as from the decision makers to design further *in vitro* and *in vivo* studies to better characterize biological actions and potential risks of marijuana and other cannabis derivatives. This is especially urging since even habitual exposure to THC appears to be capable of impacting on human cell-mediated immunity and host defense (167). Moreover, recent animal studies showed that parental or prenatal exposure to cannabis could trigger epigenetic changes that led to significant transgenerational immunological consequences (168). Indeed, perinatal exposure of mice to THC was found to trigger profound T cell dysfunction, thereby suggesting that children of marijuana abusers who have been exposed to THC *in utero*, may be at a higher risk of exhibiting immune dysfunction and contracting infectious diseases including HIV infection (169). Following up the line of the possible long-term consequences of marijuana consumption, it is important to note that although acute THC exposure in adolescent mice is anti inflammatory, it also has long-lasting proinflammatory effects on brain cytokines, and this modulation may affect vulnerability to immune and behavioral diseases in adulthood (170, 171).

Intriguingly, in spite of the above data, in an early double-blind, placebo-controlled human study no endocrine or immunological alterations were observed upon THC use (172). However, a more recent study, which aimed to assess the effects of medical cannabis ingestion on peripheral blood mononuclear cells, revealed an immunosuppressive effect of cannabinoid preparations *via* deactivation of signaling through the proinflammatory p38 MAP kinase and mTOR pathways and a concomitant deactivation of the promitogenic p42/p44 extracellular signal-regulated kinase (ERK)-1/2 signaling. However, it should also be noted that long-term cannabis exposure in two patients resulted in reversal of this effect (173). Similar to these data, a significant decrease in serum immunoglobulin (IgG and IgM) levels, in C3 and C4 complement protein concentrations, as well as in absolute numbers of T and B lymphocytes and natural killer (NK) cells was observed in bhang (an edible form of cannabis) users as compared to controls. Interestingly, FAAH (the major eCB-degrading enzyme) expression also showed significant decrease in lymphocytes of these subjects (174).

## Open Questions, Future Challenges, and Perspectives

Although research efforts of the last three decades provided an extremely large (and ever increasing) body of evidence, there are still significant gaps in our understanding with respect to the cannabinoid signaling, and its optimal therapeutic exploitation, inviting obviously a number of specific complementary *in vitro, in vivo* and clinical studies. Along these lines, several important challenges should be faced and handled.

### Potential Side Effects

From the point-of-view of future drug development, the most obvious challenge is to avoid potential psychotropic and cardiac side effects, as well as development of tolerance and dependence due to activation of CB_1_ [overviewed in Ref. (8)]. Moreover, administration of THC and activation of CB_1_ were shown to lead to memory impairment, most probably due to the activation of the recently discovered, mitochondrially expressed subset of the receptor (175, 176), suggesting that extracellularly restricted CB_1_ agonists may be devoid of such side effects. Development of such compounds can therefore be a promising future direction in cannabinoid-based experimental pharmacology. Interestingly, however, it should also be noted that memory-impairing effect of CB_1_ activation appears to be age-dependent; in fact, THC CB_1_-dependently *improved* memory function in aged mice (177). Furthermore, by using CB_1_^−/−^ mice, it has also been demonstrated that lack of homeostatic CB_1_ signaling leads to a premature decline in cognitive abilities (178), and chronic THC administration-induced dramatic and sustained downregulation of CB_1_ was also suggested to play a role in cannabis-induced cognitive dysfunction (179). Altogether, these data clearly indicate that memory problems can occur on the basis of both overactivation and critical impairment of CB_1_ signaling. Considering the aforementioned, somewhat confusing data, focused studies are definitely required to exclude potential memory-impairing side effects of any future brain-penetrating CB_1_ agonists before their clinical administration.

On the other hand, we should also keep in mind that antagonism/inverse agonism of CB_1_ located in the CNS can also lead to serious neuropsychiatric side effects (including suicide), as it became evidenced by the infamous, brain-penetrating CB_1_ inverse agonist rimonabant (SR141716; trade names: Acomplia and Zimulti), which was applied as a potent anorexigenic agent for a few years in Europe, but was then retracted from the market (180). Fortunately, keeping CB_1_ modulators out from the CNS can relatively easily be solved by designing peripherally restricted molecules, which cannot penetrate through the blood-brain barrier.

It should also be mentioned that in a recent phase 1 trial administration of a FAAH-inhibitor named “BIA 10-2474” led to the death of one volunteer and produced mild-to-severe neurological symptoms in four others (181, 182). Importantly, it has been proven that BIA 10-2474 was a very unspecific, promiscuous lipase inhibitor, and that fatal side effects most probably developed due to a complex metabolic dysregulation in the CNS caused by the inhibition of some of its off-targets, which underscores the importance of rigorous preclinical testing of any drug candidates which are planned to be applied in human studies.

### Variable Composition of Cannabis-Derivatives, Impact of Cannabis Use History of the Patients

Clinical efficiency of complex cannabis-derivatives may greatly depend on their exact composition (115), since beyond the pCBs (each of which already possesses remarkably complex, unique molecular fingerprint), they contain many other biologically active, non-pCB components as well. Therefore, it is crucially important to describe biological actions and identify cellular targets of these so far neglected components in well-controlled future studies.

Unfortunately, rigorous assessment of pCBs’ clinical efficiency is complicated by several factors. Indeed, in a recent study, Scott et al. found that the combination of THC and CBD was more effective in killing HL60 leukemia cells than the individually applied pCBs. Even more importantly, using cannabinoids *after* the chemotherapy resulted in greater induction of apoptosis (183), highlighting that even the schedule of administration may influence the measured efficiency.

Another important issue which should be kept in mind while interpreting results of studies involving cannabis users is how well-controlled and reliable those prospective and retrospective human studies are. First, self-admission about the history of marijuana consumption may be misguiding. Second, due to the aforementioned “transgenerational” effects (168–171), in an “ideal” clinical study, inclusion/exclusion criteria should also consider “family history” of marijuana consumption. Third, purity/quality of the self-administered marijuana, as well as exposure to other illicit drugs, to alcohol (184), or to drugs belonging to the “gray zone,” e.g., *novel psychoactive substances* [NPS, a.k.a. “designer drugs”; synthetic, psychoactive substances that are generally not (yet) under international regulatory control, and among which several synthetic cannabinoids are now present at the black market (185)] should also be explored, since these all can deeply influence immunological effects of acutely applied pCBs, thereby falsifying the results [e.g., acute application of pCBs was found to significantly inhibit both the basal and C-C motif chemokine ligand 2 (CCL2)-stimulated migration of monocytes, but only in individuals non-naive to *Cannabis* (186)].

## Concluding Remarks—Lessons to Learn from *Cannabis*

Research efforts of the past few decades have unambiguously evidenced that ECS is one of the central orchestrators of both innate and adaptive immune systems, and that pure pCBs as well as complex cannabis-derivatives can also deeply influence immune responses. Although, many open questions await to be answered, pharmacological modulation of the (endo)cannabinoid signaling, and restoration of the homeostatic eCB tone of the tissues augur to be very promising future directions in the management of several pathological inflammation-accompanied diseases. Moreover, in depth analysis of the (quite complex) mechanism-of-action of the most promising pCBs is likely to shed light to previously unknown immune regulatory mechanisms and can therefore pave new “high”-ways toward developing completely novel classes of therapeutic agents to manage a wide-variety of diseases.

## Author Contributions

All authors wrote, edited, and approved the final version of this manuscript.

## Conflict of Interest Statement

The authors declare that the research was conducted in the absence of any commercial or financial relationships that could be construed as a potential conflict of interest.
